# Synopsis of the genus *Raphia* P. Beauv. (Arecaceae, Calamoideae) in the Republic of the Congo

**DOI:** 10.3897/phytokeys.271.174506

**Published:** 2026-02-10

**Authors:** Sydney T. Ndolo Ebika, Gaël U. D. Bouka, David J. Harris, Raymond G. Elenga

**Affiliations:** 1 Faculté des Sciences et Techniques, Université Marien Ngouabi, Av. des 1ers Jeux Africains, B.P. 69, Brazzaville, Congo Université Marien Ngouabi Brazzaville Congo; 2 Initiative des Champignons et des Plantes du Congo, Immeuble en diagonal du Cristal Hotel, 2è étage, Manianga, Djiri, B.P. 2300, Brazzaville, Congo Royal Botanic Garden Edinburgh Edinburgh United Kingdom; 3 Royal Botanic Garden Edinburgh, 20a Inverleith Row, Edinburgh EH3 5LR, UK Initiative des Champignons et des Plantes du Congo, Immeuble en diagonal du Cristal Hotel Brazzaville Congo

**Keywords:** Congo River Basin, ecology, ethnobiology, fibres, measurement point of palm petiole

## Abstract

*Raphia* is a genus of palm belonging to the family Arecaceae composed of 22 species. Recent floral treatments of the genus have been published for the Central African region but data on species from the Republic of the Congo is lacking. In addition, it has been suggested in those treatments that more data should be collected in the field to refine the IUCN assessments of *Raphia* species such as *R. matombe* and *R. rostrata*. Taxonomically, species delineations and distribution ranges are still somewhat incompletely known for *R. hookeri*, *R. laurentii*, *R. monbuttorum* and *R. sese*. Thus, this paper aims to provide new data about *Raphia* species occurring in the Republic of the Congo and addresses some of the knowledge gaps in the genus. We propose a new measurement point for the diameter of the petiole that we refer to as DP30 to standardize comparison within the genus. We collected herbarium specimens and took high-quality photographs in nine departments of the Republic of the Congo. We measured leaves and leaflets on mature individuals bearing either inflorescences, infructescences, or both. Information on the use of materials from *Raphia* species is presented. We record seven *Raphia* species from the country: *R. gentiliana*, *R. hookeri*, *R. laurentii*, *R. matombe*, *R. regalis*, *R. rostrata* and *R. textilis*. The first two species have a wide distribution range across the country whereas *R. matombe* is only recorded in two departments and *R. regalis*, *R. rostrata* and *R. textilis* are recorded in only one department. *R. hookeri* and *R. laurentii* are the only two species which form large mono-dominant stands in swamps in the Republic of the Congo. We found that different parts of the *Raphia* species plants are used for different purposes. The petiole and leaflets are used in the construction of buildings. The pith is used to make decorative objects and toys. The epidermis of the young leaflets is used to make raffia loincloths and traditional dance skirts. The sap is tapped to make *Raphia* wine. Individuals of some *Raphia* species are cut down, wounded, and left to decay to harvest the larvae of *Rhynchophorus cf. phoenicis* (Fabricius, 1801) (Coleoptera, Curculionidae). More observations and collections from the field are needed for a better understanding of this genus in Africa.

## Introduction

The genus *Raphia* P. Beauv. contains about 22 species (Govaerts and Dransfield 2005; Stauffer et al. 2014; Kamga et al. 2018). Almost all species occur in continental Africa, with one species (*R. taedigera* (Mart.) Mart.) in Central America and Brazil, and another species (*R. farinifera* (Gaertn.) Hyl.) extending from East Africa to Madagascar (Dransfield 1986b).

*Raphia* species are very important to the economy, culture, and ecology in Africa and Madagascar. See Otedoh (1982), Brink and Achigan-Dako (2012), Mbandu Luzolawo et al. (2018), Kamga et al. (2020) and Stauffer et al. (2021) for major and minor uses of this genus.

Economically, the genus contributes in several ways to the rural economy in Republic of the Congo (MRSIT 2018). It provides building materials, textile fibres, food, *Raphia* wine, and red vegetable oil, and thus could significantly contribute to alleviating the housing and food shortage encountered in the areas in which they are found. In almost all villages, some *Raphia* species are used in construction. Even the most modern buildings in cities can have thatched *Raphia* kitchens. In many villages, all the houses may be made of *Raphia* petioles and leaflets. A large part of the rural economy is based on *Raphia* construction but because this is often carried out at the subsistence level, it is not recorded by economists, and it is rarely considered in policies on the rural economy. The second major economic activity based on *Raphia* in the Republic of the Congo is palm wine tapping. This commodity is widespread and part of the cash economy. Many people have full-time employment harvesting or selling palm wine, and it is transported to all the large towns where it is sold fresh and untreated from *Raphia hookeri* G.Mann & H.Wendl or warmed up and served hot when from *Raphia laurentii* De Wild.

Historically, fibres have been commercially important in the genus across the world. The fibres of *Raphia* can be divided into two categories. The fibres extracted from the young leaflets are still used widely in many countries and are often referred to as “raffia” (Tuley 1995; Sandy and Bacon 2001). Historically, the use of cloth woven from *Raphia* was used as a precolonial currency in the Kongo kingdom. According to Edoumba (2001), 40 × 40 cm woven squares of cloth made from *Raphia* was called *lubongo* (plural *mbongo*). The latter were used as means of commercial exchanges between Téké and Mbosi people in the *Cuvette Congolaise* region and between Téké and Kongo in the Niari valley (Niangui Goma 2022). Later, it gave the word *mbongo* for money in the present day languages of Lingala and Kituba (Kawata 2014). The second category of fibres comes from the petiole base or leaf sheaths and are released as the leaves disintegrate on the tree. These fibres have historically been referred to as “piassava” (Chevalier 1932) and were globally traded from west and central Africa until widely replaced by plastics during the 1960s. The fibres of “piassava” and other fibres in the petioles have potential to be used in several commercial applications (Elenga et al. 2011; Elenga et al. 2013).

Culturally, the genus *Raphia* plays an important role in the Republic of the Congo. In addition to the use of construction and palm wine, there is extra cultural significance that is difficult to quantify in purely economic terms. Such services related to cultural practices are considered by the UNESCO as ‘Intangible Cultural Heritage’, which includes traditions or living expressions inherited from our ancestors and passed on to our descendants, such as oral history and the knowledge and skills to produce traditional crafts. Products from *Raphia* are important to the culture of many Congolese people even if they are not used daily. For example, *Raphia* cloth and items of clothing are usually reserved for cultural events such as weddings and funerals and carry important social and religious implications (Masson Detourbet 1957).

Ecologically, the genus *Raphia* plays several roles. The most important is that *Raphia* species are dominant in many swamps across the Republic of the Congo (Dargie et al. 2017). In the north of the country, one species, *R. laurentii*, is the major component of the globally important peat swamp (Bocko et al. 2023). Sometimes the difference in ecological niche can be quite subtle. For example, *R. hookeri* and *R. laurentii* both occur in swamps in Likouala and Sangha Departments, however they only rarely occur in inter-mixed stands. In one part of a swamp, one finds one species and maybe nearby, but in a different micro-habitat, the other species. Swamps, however, are not the only habitat in which *Raphia* species occur. They also occur on *tierra firme* (non-inundated soils) and can be the most conspicuous individual plants in the landscape. This is the case for *R. regalis* Becc., in which individual plants that occur on *tierra firme* have very large leaves that come straight from the ground, allowing the palm to be recognized from satellite images. The importance of *Raphia* to the ecology of vertebrates remains understudied in the Republic of the Congo. In the Lake Télé Community Reserve, for example, Rainey et al. (2010) reported that elephants seasonally migrate into *Raphia* swamps, hornbills carry the oil rich fruit, and gorillas use *Raphia* leaves extensively to make their nests.

Taxonomically, most species were described in the colonial period and the descriptions and type specimens are fragmentary and scattered in herbaria in Europe. The descriptions were mainly based on morphological characters such as growth habit, fibres, inflorescences, and fruits. Based on such characters, Otedoh (1982) created five sections: Moniliformes, Temulentae, Raphia, Flabellatae, and Obclavatae and one subsection, Erectae, in the section Moniliformes to accommodate the *Raphia* species and infra-specific taxa. Helmstetter et al. (2020) maintained four of the sections of Otedoh, sunk one section, Flabellatae, into Moniliformes, and elevated subsection Erectae to the rank of section. Tuley (1995) provided two simplified keys to allow identification whether the plant has mature inflorescences or not. Recent studies have contributed to improving knowledge on species diversity of the genus *Raphia* in west and central Africa (Kamga et al. 2018; Mogue Kamga et al. 2019; Helmstetter et al. 2020; Mbandu Luzolawo et al. 2020; Stauffer et al. 2021; Couvreur and Sunderland 2022), with an addition of two new species, *R. gabonica* Mogue, Sonké & Couvreur and *R. zamiana* Mogue, Sonké & Couvreur, described from Gabon and Cameroon, respectively (Kamga et al. 2018).

Building on taxonomic treatments and documentation of multiple uses of this genus in other African countries, the aims of this paper are to (1) investigate the species of *Raphia* occurring in the Republic of the Congo and how they differ from each other (2) provide the means of identifying the species with a dichotomous key and descriptions of *Raphia* species and (3) provide a preliminary summary of the different local names and uses of *Raphia* species in the country.

## Methods

### Study area

**Figure 1.**
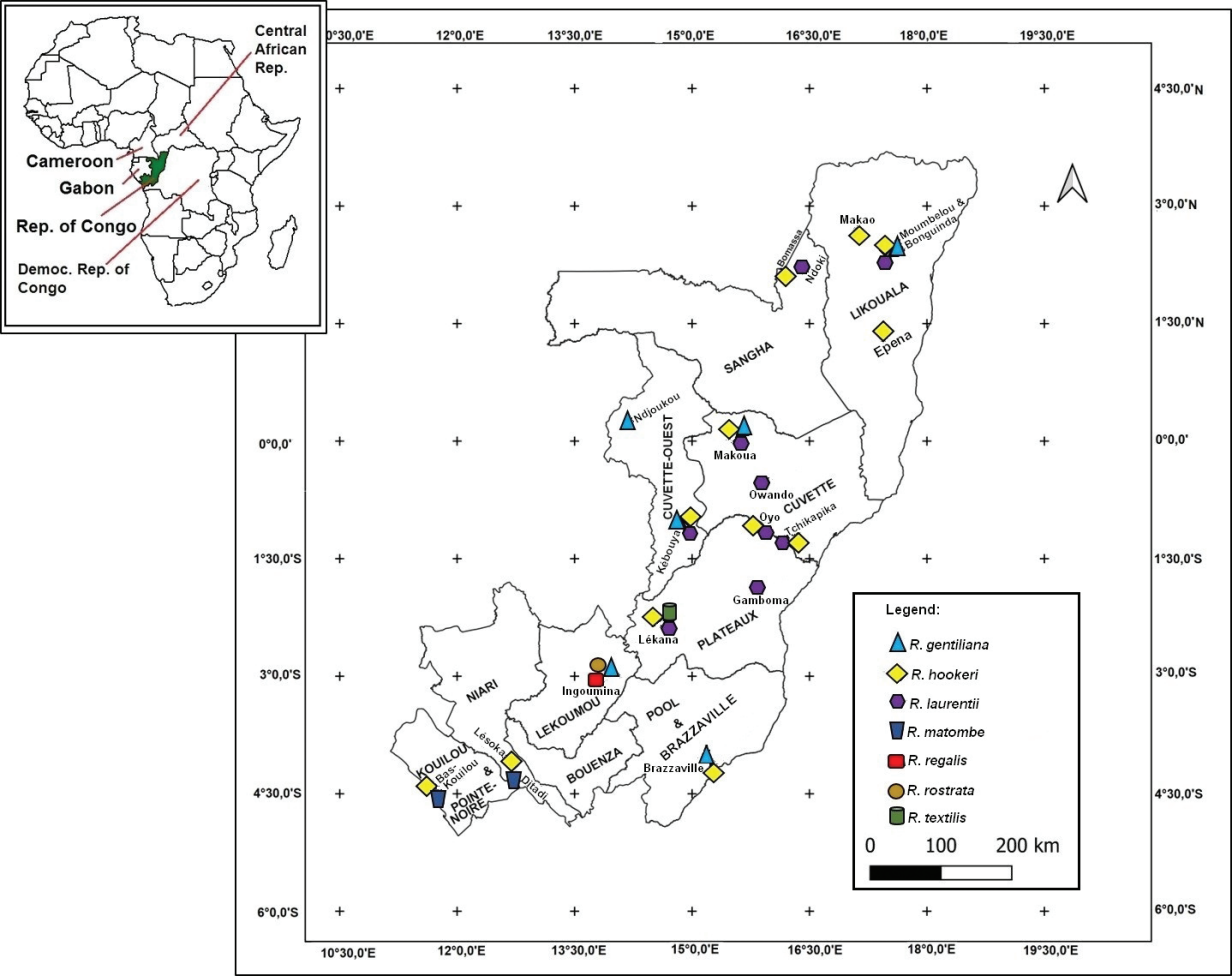
Map of the departments, collecting sites, and *Raphia* species recorded in the Republic of the Congo during our field trips.

Fieldwork was conducted in nine departments of the country, namely Brazzaville, Cuvette, Cuvette-Ouest, Kouilou, Lékoumou, Likouala, Niari, Plateaux and Sangha from 2018 to 2020 (Fig. 1).

### Special morphological characters of *Raphia* observed in the field

**Figure 2.**
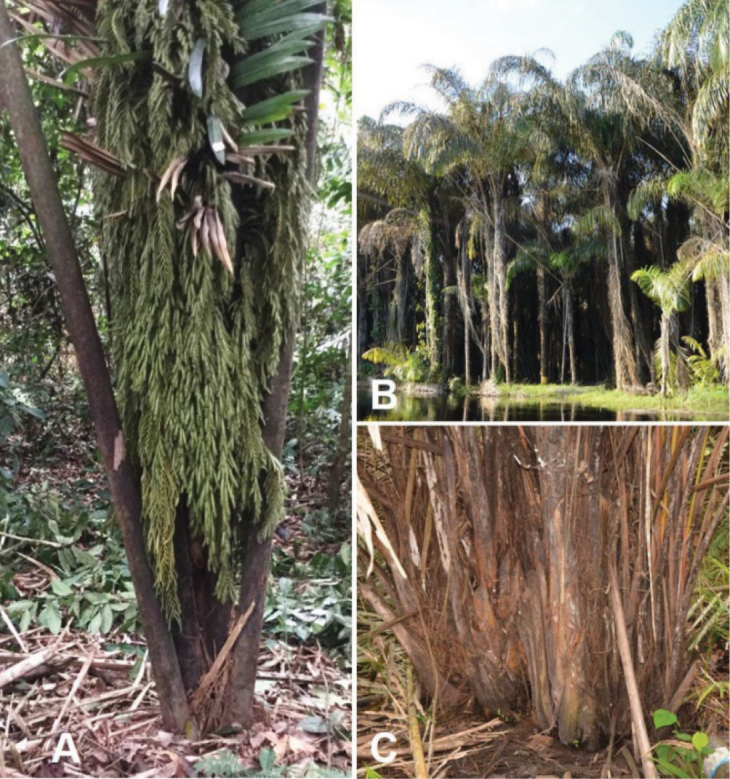
Growth habit of *Raphia* palms. **A**. Leaves arising directly from the ground level (*R. regalis*); **B**. Single stipe and gregarious (*R. hookeri*); **C**. Clustered stipes (assumed to be joined below) (*R. matombe*). **A** from *Ndolo Ebika & Harris 2753*; **B** from field observation by *Ndolo Ebika*; **C** from *Ndolo Ebika & Harris 2739*.

Growth habit: this is mostly used to characterize stipes whether they are underground or aerial, solitary or clustered (Fig. 2). The joining of stipes can be hard to observe with certainty in many cases, but if two or more stipes arise from the same place, we describe the species as clustered.

**Figure 3.**
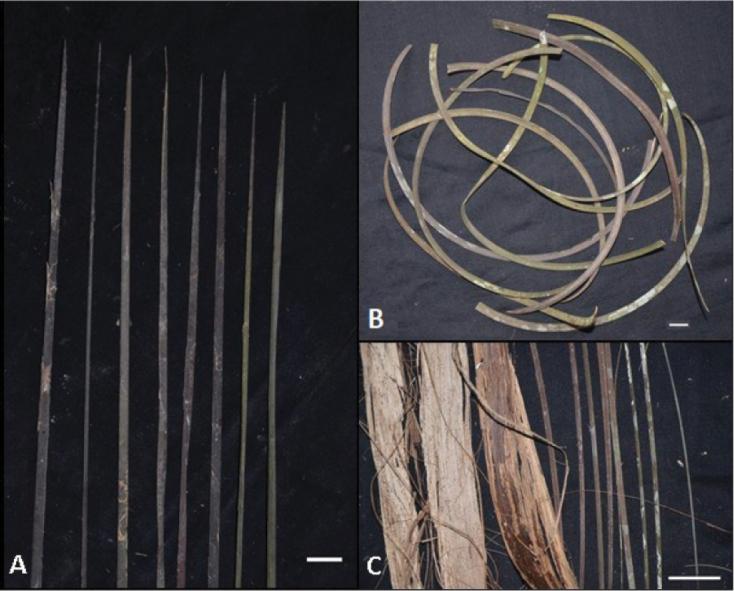
Types of fibres on the stipe of *Raphia* palms. **A**. Straight and oriented upwards (*R. laurentii*); **B**. Curling (*R. hookeri*); **C**. Belt-like fibres (left hand side of image) that disintegrate to form straight narrow hanging fibres (right hand side of image) (*R. gentiliana*). **A** from *Ndolo Ebika 2706*; **B** from *Ndolo Ebika 2707*; **C** from *Ndolo Ebika 2710*. Scale bars: 2 cm (**A, B**); 4 cm (**C**).

Stipe fibres: fibre characters used in the key are features observed around the stipe and resulting from the disintegration of the margins of the base of the petiole and the leaf sheath (Fig. 3). The size, shape, and orientation of these fibres, whether straight upwards, hanging, or strongly curling to form a coil looking like a spring, are useful in distinguishing the species.

**Figure 4.**
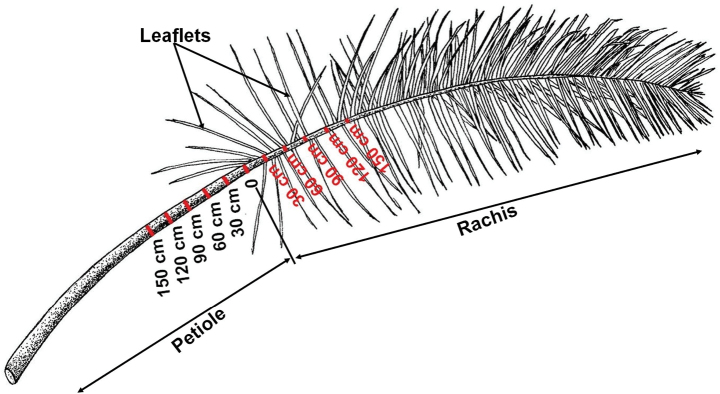
Subdivision of a *Raphia* leaf by intervals of 30 cm, from the distal point of the petiole, in two directions. Drawing by Sydney T. Ndolo Ebika.

Leaves and leaflets: from each *Raphia* species studied, five leaves in good condition were selected for measurement of the diameters of the petiole and the rachis and counting of the leaflets. On each leaf selected, the ten largest leaflets in good condition were selected for the measurement of the length and the width. To compare petiole and rachis characters across species of *Raphia*, the following system was used to standardize positions of measurements (Fig. 4). The reference point of the first leaflets on the rachis was chosen as point zero as it is usually unambiguous on any given leaf, in contrast to the petiole base which is sometimes hard to locate in the dense mass of leaf bases and fibres. Petiole diameters were made every 30 cm from the first leaflet going towards the base of the petiole. So “DP30” is the diameter of the petiole 30 cm from the first leaflet. Likewise, DR30 is the diameter of the rachis, 30 cm from the first leaflet towards the apex of the rachis. In addition to the diameter of the rachis, the number of leaflets was counted at positions along the rachis referred to as R0.30, R30.60, R60.90, R90.120, R120.150 (see Table 1).

**Table 1.** List of leaf characters used to assess morphological variation.

N°	Code	Name of Character
1	DP30	Diameter of the petiole at 30 cm below the first leaflet
2	DP60	Diameter of the petiole at 60 cm below the first leaflet
3	DP90	Diameter of the petiole at 90 cm below the first leaflet
4	DP120	Diameter of the petiole at 120 cm below the first leaflet
5	DP150	Diameter of the petiole at 150 cm below the first leaflet
6	DR30	Diameter of the rachis at 30 cm above the first leaflet
7	DR60	Diameter of the rachis at 60 cm above the first leaflet
8	DR90	Diameter of the rachis at 90 cm above the first leaflet
9	DR120	Diameter of the rachis at 120 cm above the first leaflet
10	DR150	Diameter of the rachis at 150 cm above the first leaflet
11	Total.length	Total length of the leaf (this is sheath + petiole + rachis)
12	Petiole Sheath.length	Length of the sheath and petiole
13	Rachis.length	Length of the rachis
14	Leaflet.length	Leaflet length
15	Leaflet.width	Leaflet width
16	Leaflet.aspect ratio	Ratio of the length and the width of the leaflet
17	R0.30	Number of leaflets from 0 to 30 cm on the rachis
18	R30.60	Number of leaflets from 30 to 60 cm on the rachis
19	R60.90	Number of leaflets from 60 to 90 cm on the rachis
20	R90.120	Number of leaflets from 90 to 120 cm on the rachis
21	R120.150	Number of leaflets from 120 to 150 cm on the rachis
22	DP.mean	Mean of the diameters of the petioles
23	DR.mean	Mean of the diameters of the rachis
24	PetioleVSTotal	PetioleSheath.length / Total.length as %
25	Shape	Leaflet.width/ Rachis.length: variable of the shape (per specimen)

Inflorescences, infructescences, and fruits: the length and width were also measured on different stages of the inflorescences, infructescences, and fruits. The colour of the fruit was described from fresh material.

### Herbarium specimens and data collection

Across the country, specimens were only collected from mature individuals bearing inflorescences, infructescences, or both, following recognised techniques for collecting palms (Dransfield 1986a). Random sampling was used in order to capture the full morphological variability of individuals. High quality photographs of the individuals, the leaves, the inflorescences, and the flowers or fruits were taken to illustrate each species. Field notes and GPS coordinates for each specimen were recorded. Herbarium specimens were collected and dried using a portable stove as a source of heat (Ndolo Ebika 2010). A fragment of leaflet of each individual was kept in silica gel for molecular studies. Duplicates of the specimens were deposited in the herbaria of the *Initiative des Champignons et des Plantes* (HICPC) and the National herbarium (IEC) in Brazzaville and of the Royal Botanic Garden Edinburgh (E) in Edinburgh. In the case where no herbarium specimens were sampled, we made observations by taking GPS coordinates and photographs which we refer to as occurrence records. The collected specimens were identified using Otedoh’s revision of the genus *Raphia* (Otedoh 1982), Tuley’s treatment of the Palms of Africa (Tuley 1995), the “Flore du Gabon” (Mogue Kamga et al. 2019), “Flore d’Afrique centrale” (Mbandu Luzolawo et al. 2020), and “Flore du Cameroun” (Couvreur and Sunderland 2022). We looked at material available at EIC and on short visits to BR and K. We also sent some photographs to Dr Suzanne Mogue Kamga and Dr Thomas Couvreur for identification.

### Morphometric data matrix construction

The 25 leaf characters used for measurement of *Raphia* features and for statistical tests are summarised in Table 1.

### Ethnobotanical data collection

Informal interviews were conducted with local populations (Cámara-Leret et al. 2015) and local markets were visited. No fixed questions were prepared but when at the plants, we asked the informants about the local names, languages spoken, and what the plant is used for. In addition, we made direct observations on the uses of *Raphia* by local people in the field and in the village as well as opportunistically looking at markets to see what is sold there. Such observations included long-term records we had from different parts of the country and recent (since 2018) observations made during this study.

### Data analyses

To investigate the morphological differences between species, we performed statistical tests through the software R v.4.3.0 (R Core Team 2024). First, we displayed the morphological variation of the different species using box-plots. Then, we checked whether characters significantly differed between those species using ANOVA. The application of ANOVA requires that the residuals be normally distributed and that their variance does not vary between treatments (homoscedasticity) for groups with sample sizes 15 < n < 30. This test is also used when the normality hypothesis is rejected but the population size is large (N > 30). To do this, we assessed the normality of the residuals using the Shapiro-Wilk test (Royston 1982) from the stats library (R Core Team 2013). Homoscedasticity was verified using the Bartlett test (Bartlett 1937).

However, no variable in our dataset met all the conditions for applying ANOVA. Consequently, we opted to use nonparametric tests. We used the Kruskal-Wallis test, and Dunn post-hoc multiple comparison test after Kruskal-Wallis tests to compare each pair of species. We attributed different letters to identify species differing significantly for each character. For each character, two means which share the same letter are not significantly different and two means which do not share the same letter are significantly different.

We then performed a principal component analysis (PCA) via the dudi.pca function of the ade4 package to identify the main variables that discriminate species. To make the PCA graphs easier to read and avoid correlated variables, we used the means for the DP and DR variables. However, these two variables (DP.mean and DR.mean) and two others, Total.length and Rachis.length are very closely correlated, so we eliminated Rachis.length which is already expressed in the variable PetioleVSTotal and DP.mean and DR.mean by DPR.mean which presents the average of these two variables. We represented the results of the PCA on a factorial map. Then we carried out an ascending hierarchical classification (HCA) of the species on the basis of the matrix obtained through calculation of Euclidean distance between points in the PCA factorial space using the dist.dudi function. This classification was performed with the hclust function of the stat package using the Ward.D2 aggregation criterion of Ward’s method.

Finally, we performed linear discriminant analysis (LDA) or discriminant factor analysis with the discrimin function of the ade4 package. This analysis was used for testing hypotheses of morphological similarities or differences employing pairwise comparisons between two groups, by projecting a multivariate data set down to one dimension and maximizing separation between groups separated *a priori*. Other than being a support for PCA, discriminant analysis is used to identify variables that separate different morphological groups.

## Results

Seven *Raphia* species are recorded in the country: *R. gentiliana* De Wild., *R. hookeri* G.Mann & H.Wendl., *R. laurentii* De Wild., *R. matombe* De Wild., *R. regalis* Becc., *R. rostrata* Burret and *R. textilis* Welw.

### Morphometric results

**Figure 5.**
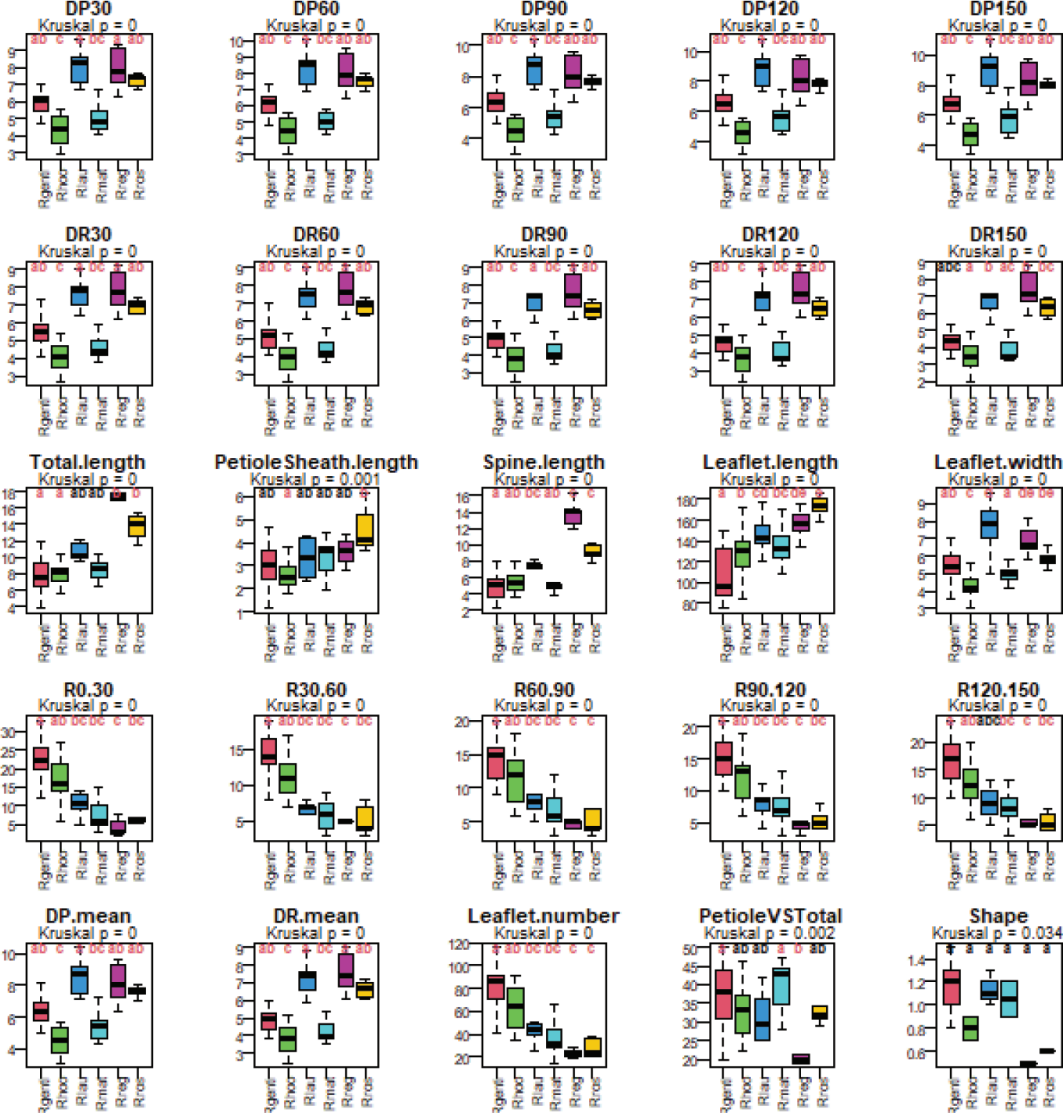
Box plots showing the variation of each character within the seven species of *Raphia*. See Table 1 for a description of each character. The black bold line represents the median value. The upper and lower sides correspond to the 3^rd^ (0.75) and 1^st^ (0.25) quartiles. The small horizontal line connected to the quartile by dashes (- - -) is placed at a maximum of 1.5 times the interquartile and visualizes the range of the variable.

The boxplots in Fig. 5 show the morphological variation of each character within each species. A Kruskal-Wallis test performed on all species in this study confirmed that all variables measured showed significant differences among species (P < 0.05 for all variables). The post hoc multiple comparison test performed after the Kruskal-Wallis test to compare each pair of species showed that most species differ from each other on at least 20 of the 25 variables. On four variables, PetioleSheath.length, PetioleVSTotal, R120.150, and Total.length, we note at least one species for which the differences are not significant with the others (variable in black in Fig. 5). These are the species *R. gentiliana*, *R. laurentii*, *R. matombe*, and *R. regalis* for the PetioleSheath.length character; the species *R. hookeri*, *R. laurentii*, and *R. rostrata* for the PetioleVSTotal character; the species *R. laurentii* species for the R120.150 character, and *R. laurentii* and *R. matombe* species for the Total.length character. For the Shape variable, the differences between all species are not significant.

**Figure 6.**
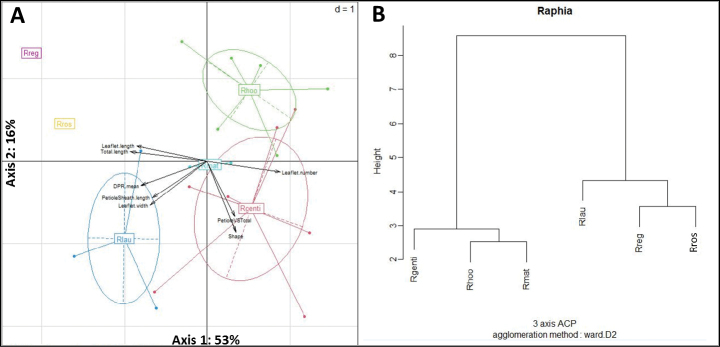
**A**. Distribution of species and variables on axes 1 and 2 of the PCA; **B**. Dendrogram of the hierarchical cluster analysis (HCA) on the basis of the matrix obtained through calculation of Euclidean distance between points in the PCA factorial space. The scale on the left corresponds to the inertia translating the distance of similarity between each class.

The first three PCA principal coordinates explain 92% of the total variance (53%, 23%, and 16% for each axis). In general, the first axis and the third axis mostly express the variation of the length variables, and the second axis expresses the variation of the other variables, notably the width, the average, and the shape (Fig. 6A, Table 2).

**Table 2.** Contribution of variables and species on the first three axes of the PCA.

Variables & species	Axis1	Axis2	Axis3
Total.length	20.86	0.71	0.43
PetioleSheath.length	10.59	10.94	24.26
Leaflet.length	17.51	1.87	2.11
Leaflet.width	11.35	15.69	15.14
Leaflet.number	18.80	0.89	3.28
PetioleVSTotal	2.62	24.61	33.11
Shape	2.91	40.66	6.93
DPR.mean	15.31	4.70	14.72
*R. gentiliana*	26.51	38.26	43.92
*R. hookeri*	17.33	29.45	26.55
*R. laurentii*	18.05	22.63	14.87
*R. matombe*	0.62	0.02	14.80
*R. regalis*	22.59	8.56	1.17
*R. rostrata*	14.86	1.05	7.50

In bold = the variables and species having greater contribution on each axis.

The following variables: Total.length, Leaflet.length, Leaflet.number, and to some extent DPR.mean, have the greatest contribution on axis 1. They discriminate *R. gentiliana* from *R. regalis* (Fig. 6A). *R. regalis* has the highest maximum values for these variables and *R. gentiliana* the lowest minimum values. Axis 2 is influenced by the variables PetioleVSTotal and Shape. These variables have the greatest contribution on this axis and allow the separation of the *R. laurentii* species from the others. *R. laurentii* has the highest maximum values for these variables. The variables PetioleSheath.length, Leaflet.width, and PetioleVSTotal have the largest contributions on axis 3. These variables isolate the species *R. matombe* and *R. rostrata*. *R. rostrata* has the highest maximum values for the variables PetioleSheath.length, Leaflet.length. The species *R. matombe* has a greater value than *R. gentiliana* for the variable PetioleSheath.length. The *R. hookeri* species, for its part, differs from other species on the three axes where the values of its contribution are respectively close.

The dendrogram (Fig. 6B) obtained following the hierarchical classification shows two branches. The first branch brings together species such as *R. gentiliana*, *R. hookeri*, and *R. matombe*. On this first branch, *R. hookeri* and *R. matombe* are closer than they are to *R. gentiliana*. The second branch of this dendrogram consists of *R. laurentii*, *R. regalis*, and *R. rostrata*. The species *R. regalis* and *R. rostrata* are closer compared to *R. laurentii*.

**Figure 7.**
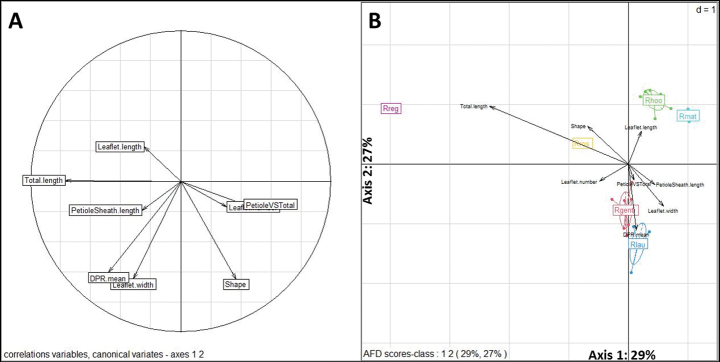
**A**. Correlation circle between variables used for LDA; **B**. Distribution of species and variables on axes 1 and 2 of the LDA.

The first three LDA principal coordinates explain 80% of the total variance (29%, 27%, and 24% for each axis). The variables Total.length (total leaf length), Leaflet.number (leaflet ratio = length/width), Shape and PetioleSheath.length (sheath and petiole length) make the greatest contribution to axis 1, separating *Raphia regalis* and *R. rostrata* from the other species. Although both species have the lowest values for these variables (except for PetioleSheath.length), *R. regalis* has even lower values for both. Axis 2 is represented by the leaf length and width variables (Leaflet.length and Leaflet.width), and the average rachis and petiole measurement (DPR.mean). This axis separates the *R. gentiliana*, *R. laurentii*, and *R. hookeri* species from the others. For the variables of axis 2, *R. gentiliana* and *R. laurentii* have low values, unlike *R. hookeri*, which has high values. Axis 3, represented by the PetioleVSTotal variable (PetioleSheath.length/Total.length), characterises *R. matombe* (Fig. 7, Table 3).

**Table 3.** Contribution of variables and species to discrimination on the first three axes of the LDA.

Variables	DS1	DS2	DS3
Total.length	2.33	0.97	0.26
PetioleSheath.length	0.44	0.34	0.44
Leaflet.length	0.22	0.55	0.17
Leaflet.width	0.59	0.70	0.41
Leaflet.number	0.48	0.28	1.34
PetioleVSTotal	0.09	0.26	0.31
Shape	0.68	0.63	0.37
DPR.mean	0.14	1.10	0.54
*R. gentiliana*	0.54	5.08	4.50
*R. hookeri*	2.56	6.46	5.53
*R. laurentii*	0.49	4.00	3.29
*R. matombe*	2.01	1.63	2.93
*R. regalis*	3.98	0.93	0.49
*R. rostrata*	0.77	0.35	0.91

In bold = the variables and species having greater contribution on each axis.

### Key to the species of *Raphia* known from the Republic of the Congo

**Table:** 

1	Plant with an underground stipe; leaves arising straight from the ground	***R. regalis***
–	Plant with an aerial stipe, sometimes inconspicuous due to dense leaf bases; leaves inserted either at the base or at the top of the aerial stipe and not emerging straight from the ground	**2**
2	Palm clustered with at least two stipes joined at the base; palms always growing in inundated places	**3**
–	Palm solitary without stipes joined at the base; palms growing either on *tierra firme* or in inundated places	**5**
3	Stipe fibres straight and oriented upwards, sharp at the apex	***R. laurentii***
–	Stipe fibres curling and hanging, not sharp at the apex	**4**
4	Petiole green, yellow or orange when still attached to the stipe; stipe fibres belt-like, up to 6 cm wide and breaking down to form small fibres 3–4 mm wide; bracts bearing a pointed tip reaching 6 cm long at the base of the partial inflorescences	***R. matombe***
–	Petiole reddish, black with white dots to green when still attached to the stipe; stipe fibres not forming a belt-like structure, at least 8 mm wide; bracts inconspicuous at the base of the partial inflorescences	***R. rostrata***
5	Fibres tightly coiled looking like springs around the stipe and between leaf bases; petiole diameter 30 cm below the first leaflet (DP30) 2.9–5.6 cm	***R. hookeri***
–	Fibres curling and hanging, forming belt-like ribbons up to 4.5 cm wide and breaking down to form smooth fibres 5–6 mm wide; petiole diameter 30 cm below the first leaflet (DP30) 4.7–7.7 cm	**6**
6	Fruits ellipsoid, rounded to cuneate at top	***R. gentiliana***
–	Fruits globose, truncate at top	***R. textilis***

### Synopsis of the genus in the Republic of the Congo

In this section, a synopsis of each species is presented. The descriptions are based on characters observed during fieldwork. The traditional taxonomic format has not been followed as the descriptions only reflect what was found in the field and did not include data from all the herbarium specimens across the species range as would be found in a taxonomic revision.

#### Raphia gentiliana

Taxon classificationPlantaeArecalesArecaceae

De Wild. (De Wildeman 1905: 29)

7325A3D4-6D48-597A-AE89-275B5C0219C8

Figs 8, 9

##### Description.

***Palms*** 6 to 10 m high, 16 cm in diameter, isolated or gregarious and forming in the latter case small mono-dominant stands of up to 17 individuals within a radius of 50 m. ***Stipes*** solitary, with two types of fibres: the narrower, rounded fibres 2–3 mm in diameter that run around the stipe and the flattened fibres, curling and hanging, forming belt-like ribbons up to 4.5 cm wide breaking down to form smooth fibres 5–6 mm. ***Leaves*** 7.60–10.58 m long. Petiole laterally fluted from base to rachis, unarmed, 3.72–4.10 m long; DP30 comprises between 4.7–7.7 cm. Rachis 6.18–6.86 m long, laterally fluted, with a row of spines on each side of the cleft. Base of rachis uneven, with an offset of 12 to 27.5 cm between the two groups of basal leaflets on either side. Basal leaflets, on both sides of the rachis, grouped into a tuft of 4–8 leaflets, 1.33–1.41 m × 8.0–8.4 cm, 12–35 in the R0-30 area of the rachis, with dorsal spines (on the main vein) 5 mm long and marginal spines 4 mm long. ***Inflorescences***: when young, 5–6 per individual, hanging, very tapered, approx. 2 m long; when mature 3.1 m (including peduncle 70 cm) long and 50–60 cm wide. Partial inflorescence 60 × 35 cm. Secondary and final branches 45 cm long. ***Infructescence*** hanging, 3.80 m long. ***Fruits*** ellipsoid to almost cylindrical, 4.8–7 × 2.5–5.5 cm, dark green when immature, becoming brownish and reddish when mature, rounded at the top with a 5 mm long beak.

**Figure 8.**
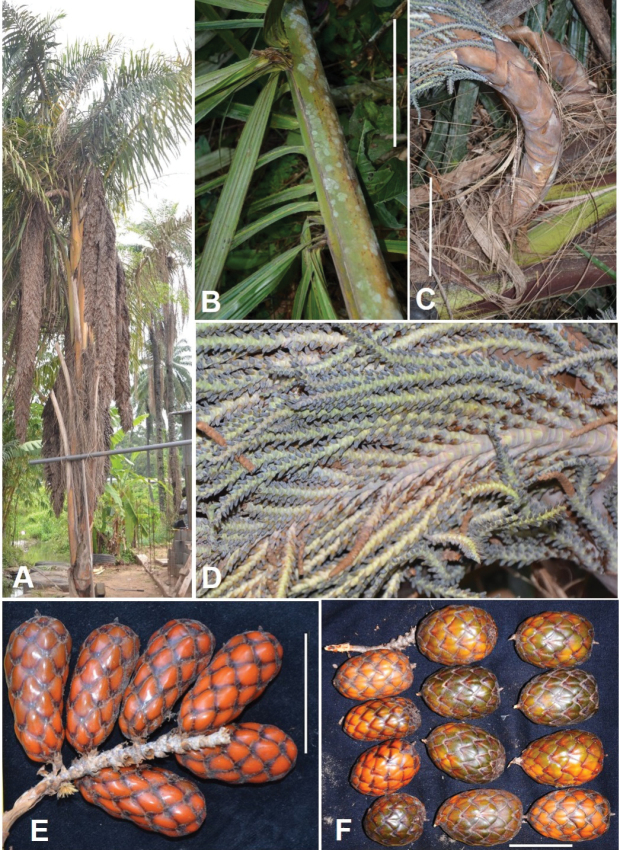
Morphology of *Raphia gentiliana*. **A**. Growth habit, with the longest infructescence (3.80 m long); **B**. Basal part of a leaf rachis; **C**. Peduncle of the inflorescence; **D**. Rachillae of a young inflorescence; **E**. Mature fruits; **F**. Immature and mature fruits. **A, E** from *Ndolo Ebika 2698***B** from *Ndolo Ebika 2458***C, D** from *Ndolo Ebika 2710***F** from *Ndolo Ebika 2726*. Scale bars: 20 cm (**B, C**); 5 cm (**E, F**).

**Figure 9.**
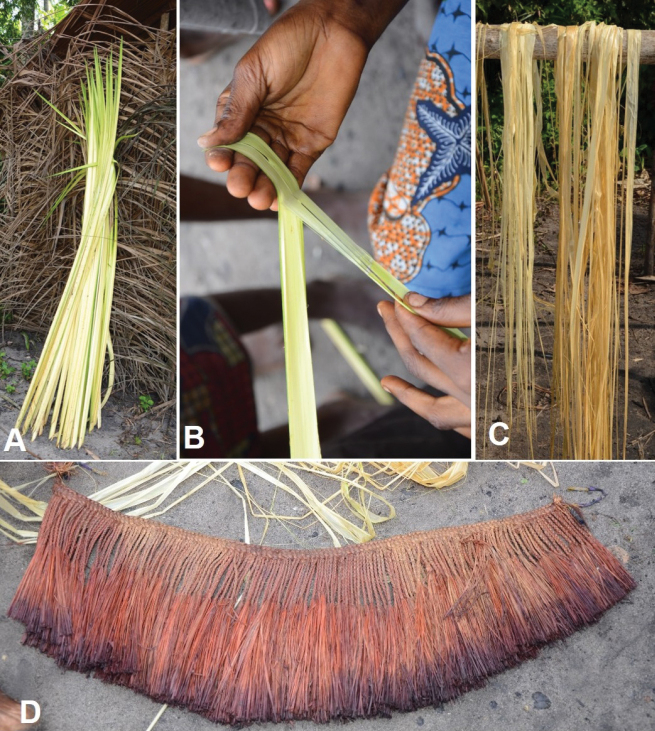
Use of leaflets of *Raphia gentiliana*. **A**. Bunch of young leaflets recently harvested; **B**. Extraction of the leaflet epidermis (raffia fibre) in Moumbelou; **C**. Sun drying of the epidermis in Moumbelou; **D**. Traditional dance skirt dyed red in Moumbelou.

##### Ecology.

Mainly growing on *tierra firme* in secondary forest, in light gaps, old fields, edge of roads, and swamps, planted in villages. It flowers in February, August, and October, and fruits in May, August, and October.

##### Distribution.

Angola, Central African Republic, and Democratic Republic of the Congo (Mbandu Luzolawo et al. 2020), Republic of the Congo (this study); in the Republic of the Congo, the species occurs in Brazzaville, Cuvette, Cuvette-Ouest, Lékoumou, Likouala, and Plateaux.

##### IUCN conservation assessment.

*Raphia gentiliana* has been assessed as Least Concern (LC) by Cosiaux *et al*. (2018a) who recommend further studies to better understand the area of occupancy of the species.

##### Uses.

The epidermis of the young leaflets of *Raphia gentiliana* is used to make traditional dance skirts in Moumbelou and Bonguinda (Likouala). The sap is tapped and used to make *Raphia* wine called Pigui in Ingoumina (Lékoumou). The petiole is used as laths to make the framework of huts, the skeleton of hut walls, and to make the two sticks that make up the skeleton of roofing tiles. In Kébouya (Cuvette-Ouest), the palm tree is felled, wounded at several points, and left to decay in order to harvest the edible larvae of *Rhynchophorus cf. phoenicis*.

##### Vernacular names.

Pigui, Pih-ongoungouma in Téké (Cuvette-Ouest); Opeou in Makoua, (Cuvette); Pigui in Téké (Lékoumou); Pòndo or Dihondò in Mbenzele (Likouala).

##### Notes.

*Raphia gentiliana* resembles *R. textilis* in the palm growth habit and the use of the epidermis of the young leaflets. We found that the easiest character to distinguish the two species is the fruit shape. Fruits are ellipsoid with a rounded to cuneate apex in *R. gentiliana*, while globose with a truncate apex in *R. textilis*. Tuley (1995) described the fruits as variable in shape, tapering club shaped to more globose or ellipsoid/ovoid for *R. gentiliana*, turbinate and sometimes near cylindrical in shape for *R. textilis*. Mbandu Luzolawo et al. (2020) reported the use of the epidermis of the young leaflets of *R. gentiliana* in the Democratic Republic of the Congo whereas Mogue Kamga et al. (2019) reported a similar use for *R. textilis* in Gabon. Further investigations in the field are needed for a better understanding of the two species.

##### Specimens examined.

**Republic of the Congo**. • Brazzaville. Saint Denis, 04°06.41'S, 015°12.78'E, 332 m, 27 Oct 2018, *Ndolo Ebika 2458* (HICPC); Mfilou, 04°11.26'S, 015°17.35'E, 372 m, 17 May 2019, *Ndolo Ebika 2698* (HICPC); • Cuvette. Makoua, 00°00.0'N, 015°36.13'E, 338 m, 22 Aug 2019, *Ndolo Ebika 2734*, (HICPC); • Cuvette-Ouest. Kébouya, 01°08.65'S, 014°56.79'E, 394 m, 17 Aug 2019 *Ndolo Ebika 2710* (HICPC); 01°08.75'S, 014°56.62'E, 425 m, 18 Aug 2019, *Ndolo Ebika 2726* (HICPC); Ndjoukou. 00°12.58'N, 014°10.56'E, 539 m, 16 Feb 2020, *Ndolo Ebika 3079* (HICPC); • Lékoumou. Ingoumina, 7 km from Zanaga on the road to Sibiti and 1.7 km E from the village Ingoumina, 02°53.48'S, 013°51.81'E, 570 m, 1 Oct 2019, *Ndolo Ebika & Harris 2754* (HICPC, E); 7 km from Zanaga on the road to Sibiti, 02°52.51'S, 013°50.06'E, 540 m, 1 Oct 2019, *Ndolo Ebika & Harris 2758* (HICPC, E); • Likouala. Moumbelou, 02°24.40'N, 017°32.04'E, 345 m, 15 Aug 2018, *Ndolo Ebika s.n*. [photo only] (HICPC).

#### Raphia hookeri

Taxon classificationPlantaeArecalesArecaceae

G.Mann & H.Wendl. (Mann and Wendland 1864: 24: 438)

40041251-487C-562A-93AC-E8510A613052

Figs 10, 11

##### Description.

***Palms*** 6–8 m high, gregarious with several individuals forming a monodominant stand. ***Stipes*** solitary, rarely appearing clustered, fully covered with fibres forming dense springs; fibres at least 8 mm wide. ***Leaves*** 5.65–10.5 m long. Petiole 1.81–3.80 m long, not spiny, channeled on the upper side (that facing the stipe); DP30 comprised between 2.9–5.6 cm. Rachis 3.60–7.92 m long; channeled on the upper side, spiny on each side of the cleft. Leaflets 85–172 × 2.6–5.8 cm; 6–27 in the R0–30 area of the rachis, with spines in two rows on the midrib and with marginal spines. ***Inflorescences*** (3–) 4–5 per individual, 1.6–1.9 m long (including a peduncle of c. 30 cm) and 57–86 cm wide. Partial inflorescences 40–60 cm long and 20–35 cm wide. Second order rachillae up to 17–34 cm long and 8 mm wide. ***Infructescence*** hanging. ***Fruits*** ellipsoid to oblong, 4.4–8.2(–10.6) × 3.1–4.9 cm, green when immature and yellow to reddish when mature, with a 1–1.5(–1.8) cm long beak.

**Figure 10.**
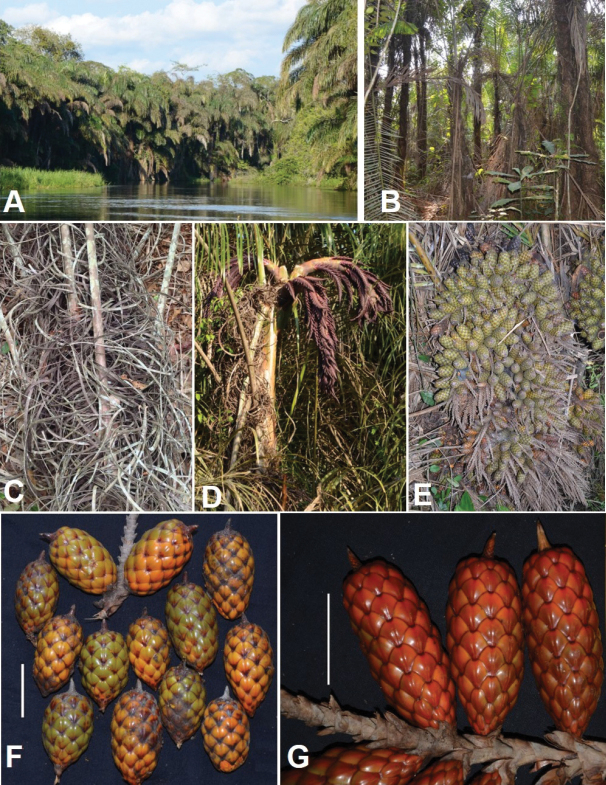
Morphology of *Raphia hookeri*. **A**. View of a ‘raphiale’ dominated by *R. hookeri* along Motaba River; **B**. Single stemmed and gregarious individuals; **C**. Curling fibres covering the stipe observed in Epena; **D**. Upper part of the stipe with young inflorescences observed along Motaba River; **E**. Infructescence with unripe fruits; **F**. Unripe and ripe fruits; **G**. Ripe fruits. **A** from field observation by *Ndolo Ebika*; **B** from *Ndolo Ebika 2707***C** from field observation by *Ndolo Ebika***E, F** from *Ndolo Ebika 2707***G** from *Ndolo Ebika 2763*. Scale bars: 5 cm (**F, G**).

**Figure 11.**
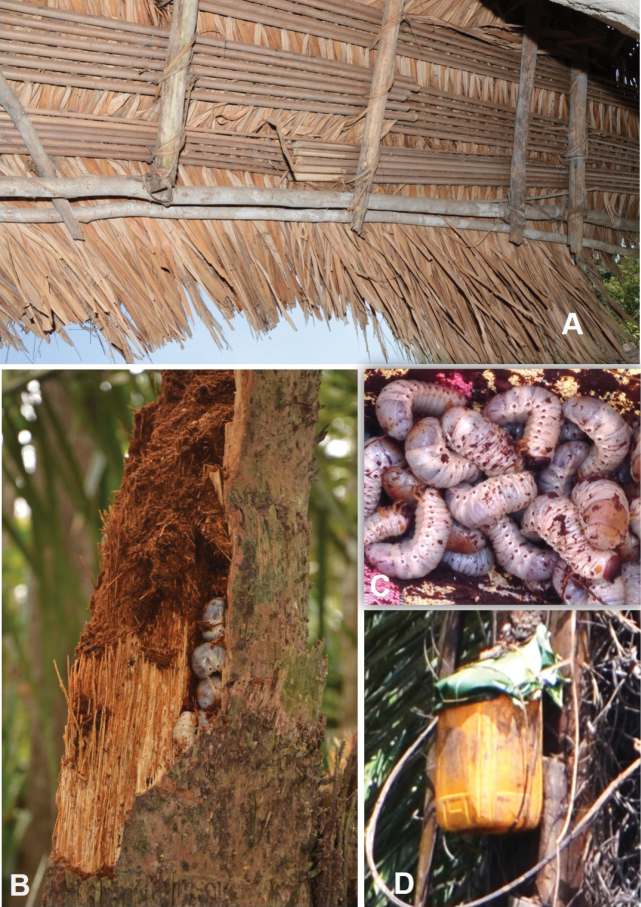
Use of *Raphia hookeri*. **A**. View from inside the house of thatch made with bundles of 5–6 leaf rachises with the leaflets still attached and on the outer side, in Moumbelou; **B**. A dead and decaying stipe containing the larvae of *Oryctes* sp. in Bas-Kouilou; **C**. Harvested larvae in Bas-Kouilou; **D**. Collection of the sap used to produce wine along the Motaba River.

##### Ecology.

Forming monodominant stands (‘raphiales’) stretching in some cases for more than 10 km, often along rivers, and planted in villages. In Republic of the Congo, it flowers in August, fruits present in May, August, September, October.

##### Distribution.

Angola (Cabinda), Benin, Cameroon, Central African Republic, Côte d’Ivoire, Democratic Republic of the Congo, Equatorial Guinea, Gabon, Gambia, Ghana, Guinea, Liberia, Nigeria, Republic of the Congo, Sierra Leone, Togo (Brink and Achigan-Dako 2012; Couvreur and Sunderland 2022; Harris 2002; Mogue Kamga et al. 2019); in the Republic of the Congo, the species occurs in Brazzaville, Likouala, Cuvette, Cuvette-Ouest, Kouilou, and Niari.

##### IUCN conservation assessment.

*Raphia hookeri* was assessed by Cosiaux et al. (2018b) as Least Concern (LC).

##### Uses.

The sap is tapped and used as *Raphia* wine called Molengue in Lingala; Péké in Mbenzele (Likouala); Mali ouliè in Téké (Lékana, Plateaux); Ouliè in Téké (Kébouya, Cuvette-Ouest); Olènguè in Mbochi (Cuvette); Ntombé in Ndzabi (Lesoka camp, Niari). The rachis, with the leaflets still attached, are grouped in 5 or 6 to cover the roofs of huts (Moumbelou, Likouala). The young leaflets are also used as cassava wrappers (Tchikapika, Cuvette). The pulp of the fruit is used to make the red oil known as “Kolo” in Mbochi (Silou et al. 2000). In Cuvette and Plateaux departments, the pulp is fermented for a few days in water after cooking, called “pandé” in Mbochi, and is eaten. The name “pandé” in Mbochi is also used for the same product from *R. laurentii*. According to Limingui et al. (2021), the fermented pulp has medicinal properties and contains proteins, fats, and sugars. The pith (inert and soft part) of young stipes called “Otowa” in Mbochi (Tchikapika and Oyo; Cuvette) can be eaten raw or cooked. People harvest the edible larvae of *Oryctes* sp. Hellwig, 1798 (Coleoptera, Scarabaeidae) from the dead and decayed stipe. These larvae are called “Aboh” (sing. Iboh) in Mbochi (Tchikapika and Oyo; Cuvette) and are sold in markets.

##### Vernacular names.

Péké in Makao (Cuvette); Péké in Mbenzele (Likouala); Ouliè, plural Iliè, in Téké (Plateaux); Olènguè, plural Elènguè, in Téké (Cuvette-Ouest), plural Ilènguè, in Mbochi (Cuvette); Bongo, plural Abongo, in Makoua (Cuvette); Ntombé in Ndzabi (Niari); Dirombi, plural Marombi, in Punu (Niari); Tohombi in Vili (Kouilou).

##### Notes.

*Raphia* species sharing the curling fibres pattern form the section Temulentae Otedoh which contained three species *R. hookeri*, *R. rostrata*, and *R. sese* De Wild. (Otedoh 1982; Tuley 1995) but now has four species with the addition of *R. gabonica* (Helmstetter et al. 2020). From our work, we found that *R. hookeri* can be distinguished from *R. rostrata* by having the fibres which form tightly coiled springs which cover the stipe whilst in *R. rostrata* the fibres are pendant and do not form tightly coiled springs. In addition, the petiole of *R. hookeri* has a smaller diameter, DP30 size is 2.9–5.6 cm, whereas the petiole is broader in *R. rostrata* which has a DP30 size of 6.7–7.7 cm. Fruits are ellipsoid to oblong with a 10–15(–18) mm long beak in *R. hookeri* whereas they are oblong to cylindrical, rarely ellipsoid; reddish, with a 8–9 mm long beak in *R. rostrata*.

*Raphia hookeri* resembles *R. sese* (not treated in this paper) by having yellow fruits in addition to the presence of the same type of fibres on the stipe. The latter species can be recognized by its fruits generally ovoid, obovoid to ellipsoid, orange to yellowish brown with a 10–18 mm beak length (Mbandu Luzolawo et al. 2020; Tuley 1995). The field observations that we made on the fruits of *R. hookeri* show an overlap in the size of the beak with reported values for *R. sese*. Such an overlap in fruits traits can lead to confusion in the identification of the two *Raphia* species. This is the case for the work by Silou et al. (2000) which was carried out on the oil called “Kolo” from *R. sese*. We believe that the species used in that study is probably *R. hookeri*, as “Kolo” is the Mbochi name given to the oil of *R. hookeri* in the Northern part of the Republic of the Congo where we collected herbarium specimens and where the oil is produced. These two observations indicate that more collections and observations should be made in the field to better understand these two taxa and find out if *R. sese* also occurs in the Republic of the Congo.

*R. hookeri* also resembles *R. gabonica* (not treated in this paper) but the latter has fruits being globose, deltoid or ovoid; orange-red fruits, with a 5 mm long beak long and grows on *tierre firme* and near streams and does not form monodominant stands (Kamga et al. 2018).

##### Specimens examined.

**Republic of the Congo**. • Brazzaville. Mfilou, 04°11.26'S, 015°17.35'E, 372 m, 17 May 2019, *Ndolo Ebika 2699* (HICPC); • Cuvette. Makoua, 00°00.10'N, 015°36.54'E, 327 m, 21 Aug 2019, *Ndolo Ebika 2729* (HICPC); • Cuvette-Ouest. Kébouya, 01°08.65'S, 014°56.79'E, 394 m, 17 Aug 2019, *Ndolo Ebika 2709* (HICPC); • Kouilou. Bas-Kouilou, 2 km NW of the bridge on the Kouilou River, West of the Bas-Kouilou village at c. 100 m away from the main road, 04°27.53'S, 011°42.30'E, 10 m, 28 Sep. 2019, *Ndolo Ebika & Harris 2742* (HICPC, E); • Likouala. Epena, 01°20.86'N, 017°26.23'E, 340 m, 07 Mar 2024, *Ndolo Ebika* s.n. [photo only] (HICPC); Moumbelou, 02°24.32'N, 017°31.87'E, 341 m, 14 Aug 2018, *Ndolo Ebika* s.n. [photo only] (HICPC); • Niari. Lesoka camp, close to Mafoubou River, about 9 km N of Dolisie, 04°08.10'S, 012°42.13'E, 320 m, 5 Oct 2019, *Ndolo Ebika & Harris 2763* (HICPC, E); • Plateaux. Lékana, 02°18.97'S, 014°32.20'E, 798 m, 14 Aug 2019, *Ndolo Ebika 2707* (HICPC).

#### Raphia laurentii

Taxon classificationPlantaeArecalesArecaceae

De Wild. (De Wildeman 1905: 26)

1EFD24B3-B5A5-5A11-BBBF-D1199CC250FC

Figs 12, 13

##### Description.

***Palms*** about 2–5 m high, gregarious with several individuals forming a monodominant stand. ***Stipes*** clustered with 2–3 stipes joined at the base. Fibres blackish, flattened, long and upward pointing, 1.60 m in length and 5–8 mm wide. ***Leaves*** intertwined from the base and obscuring the stipe, 9.46–12.20 m long. Petiole 2.35–4.25 m long, adaxially channelled from the base to almost the middle then cylindrical or fully channelled, not spiny; DP30 between 6.7–9.7 cm. Rachis 5.79–8.29 m long, cylindrical or only adaxially channelled in the first few centimetres then forming a bumped and flat ventral ridge having spines on the two sides and then converge onto a single and prominent ridge. Leaflets 1.42–1.68 m × 4.5–6.8 cm, 5–14 in the R0-30 area of the rachis, dark green above and silvery below, with marginal (on blade margin) and dorsal (on main vein) spines. ***Inflorescences*** 2–3 (–4) per individual, semi-erected when young then curving downward. ***Fruits*** broadly ellipsoid to oblong, 6.2–8.8 × 4–5 cm, green when immature and yellow-orange when mature, with a 4–7 mm long beak.

**Figure 12.**
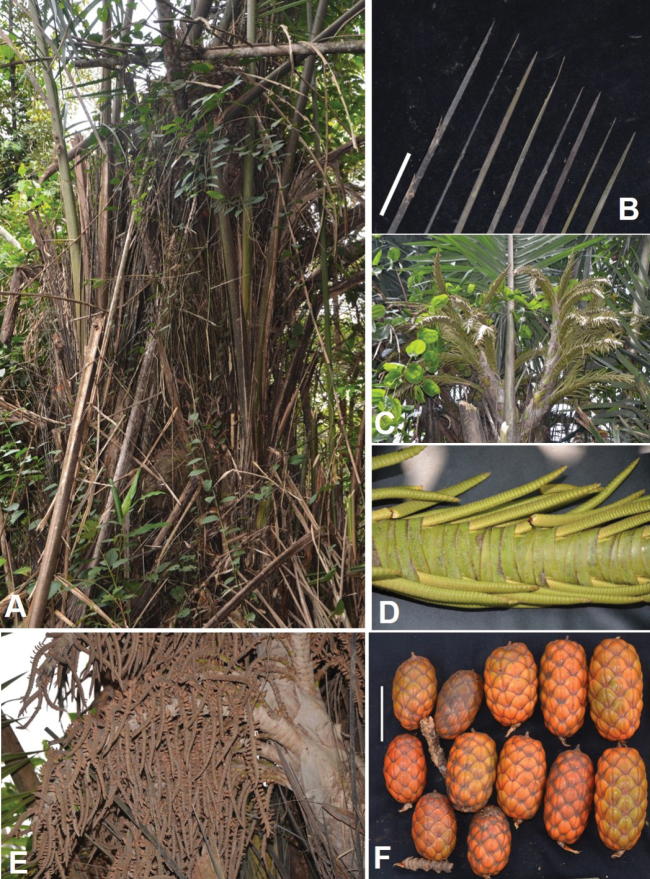
Morphology of *Raphia laurentii*. **A**. Clustering stems; **B**. Straight, pointed and upwards oriented upwards fibres; **C**. Upper part of the stipe with young inflorescences; **D**. Detail of the young inflorescence; **E**. Inflorescence; **F**. Mature fruits. **A, F** from *Ndolo Ebika 2708***B, E** from *Ndolo Ebika 2706***C, D** from *Ndolo Ebika 2728*. Scale bars: 5 cm (**B, F**).

**Figure 13.**
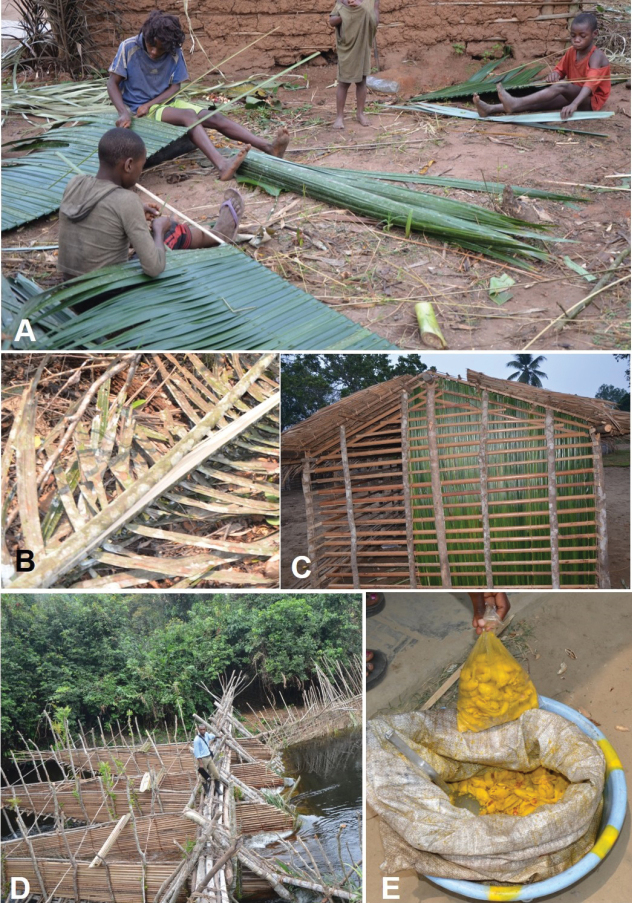
Use of *Raphia laurentii*. **A**. Making of vegetable roofing tile in Bonguinda; **B**. Collecting the hardest part of the leaf rachis; **C**. Frame of the wall made with sticks from leaf rachis then covered with leaflets, the roof framework is made of laths from petiole and covered with thatch tiles in Kébouya; **D**. Fishing trap made with petiole and rachis after removing the leaflets in Kébouya; **E**. Cooked pulp of the fruits without seeds, for sale, in Lékana. **A** from field observation by *Ndolo Ebika*; **B** from *Ndolo Ebika 2708***C–E** from field observation by *Ndolo Ebika*.

##### Ecology.

Permanently flooded swamps, in some areas in Likouala forming very large stands, mostly away from the rivers. In the Sangha Trinational it occurs close to small streams, sometimes within 100 m of *Raphia hookeri*, but on soil with a slightly lower water table than *R. hookeri*. It is cultivated in a few villages such as Lékana on *tierra firme*. In the Republic of the Congo, the species flowers and fruits in August.

##### Distribution.

Angola, Gabon, Central African Republic, Republic of the Congo, Democratic Republic of the Congo (Harris 2002; Mbandu Luzolawo et al. 2020; Mogue Kamga et al. 2019); in the Republic of the Congo: Cuvette, Cuvette-Ouest, Likouala, Plateaux.

##### IUCN conservation assessment.

*Raphia laurentii* was assessed by Cosiaux *et al*. (2018c) as Least Concern (LC).

##### Uses.

The sap is tapped and used to make *Raphia* wine called Tsam (Cuvette, Plateaux). The petiole is used as laths to make the framework of huts, and the frame of hut walls. The hardened and smooth parts of the petiole and rachis are used to make the two sticks that make up the skeleton which will be combined with the leaflets to form thatch roofing tiles. The pulp of the fruit is used to make the red oil called “Mbayaka” by the Moyis (Cuvette). Silou et al. (2000) provided details on physico-chemical characteristics and fatty acid composition of its oil. The cooked pulp is edible and is called “pandé” in Kouyou and Mbochi (Cuvette). “Pandé” is also used in Mbochi for the cooked pulp from *R. hookeri*.

##### Vernacular names.

Mabuku in Ngombe (Sangha); Baya, plural Abaya, in Téké (Cuvette-Ouest); Ivougou in Makoua (Cuvette); Ibouhou in Kouyou and Mbochi (Cuvette), plural Abouhou, in Mbochi (Cuvette); Liboungou in Mbenzele (Likouala).

##### Notes.

*Raphia laurentii* resembles *Raphia monbuttorum* Drude in the straight and pointed fibres as well as the inflorescences semi-erect when young (Couvreur and Sunderland 2022; Mogue Kamga et al. 2019). Based on inflorescences, the main difference between the two species seems to be in the second order rachillae. The second order rachillae in *R. laurentii* are digitate, flattened and 16–21 cm long whereas in *R. monbuttorum* they are non-digitate, roundish to circular and 5–7 cm long (Mogue Kamga et al. 2019). We suggest that many new collections of the different stages of inflorescence development might help clarify the situation.

##### Specimen examined.

**Republic of the Congo**. • Cuvette. Makoua, 00°00.16'S, 015°36.07'E, 338 m, 21 Aug 2019, *Ndolo Ebika 2728* (HICPC); • Cuvette-Ouest. Kébouya, 01°08.65'S, 014°56.79'E, 394 m, 17 Aug 2019, *Ndolo Ebika 2708* (HICPC); • Likouala. Bonguinda, 02°20.38'N, 017°29.10'E, 359 m, 31 Aug 2018, *Ndolo Ebika 2429* (HICPC); • Plateaux. Lékana, 02°19.25'S, 014°35.66'E, 807 m, 13 Aug 2019, *Ndolo Ebika 2706* (HICPC).

#### Raphia matombe

Taxon classificationPlantaeArecalesArecaceae

De Wild. (De Wildeman 1916: 144)

EDAB0544-9052-5129-8F81-D6E4D00C4FAA

Fig. 14

##### Description.

***Palms*** 4–5 m high, gregarious. ***Stipes*** clustered, with 2–6 stipes joined at the base. Fibres hanging, belt-like, in broad segments up to 6 cm wide, breaking down to form smooth fibres 3–4 mm wide. ***Leaves*** intertwined from the base and obscuring the stipe, 6.4–10.5 m long. Petiole yellow to pale green when young; red when mature, 1.98–4.45 m long; split from the base to almost the middle then cylindrical or fully split, not spiny; DP30 between 4.1–6.7 cm. Rachis 3.93–7.38 m long. Leaflets 108–172 × 4.2–6.6 cm, 3–15 in the R0–30 area of the rachis. ***Inflorescence*** 179 × 25 cm long. ***Infructescence*** 5–9 per individual, 145–155 × 36–48 cm (including 25 cm peduncle). Bract 25 × 15 cm, persistent, reddish brown on the inner side, folded and with acumen of 5.2–6.2 × 1.9–2.6 cm. Partial inflorescence 30–36 × 13 cm, with rachillae inserted in more than one plane, layered on top of each other. Secondary and final branches 6–8 cm long, occasionally up to 10 cm, inserted at an angle of 45 degrees with respect to the first-order branches. ***Fruits*** ellipsoid, 4.5–5 × 2.7–3 cm, green when immature and yellow at maturity, with a 5–8 mm long beak.

**Figure 14.**
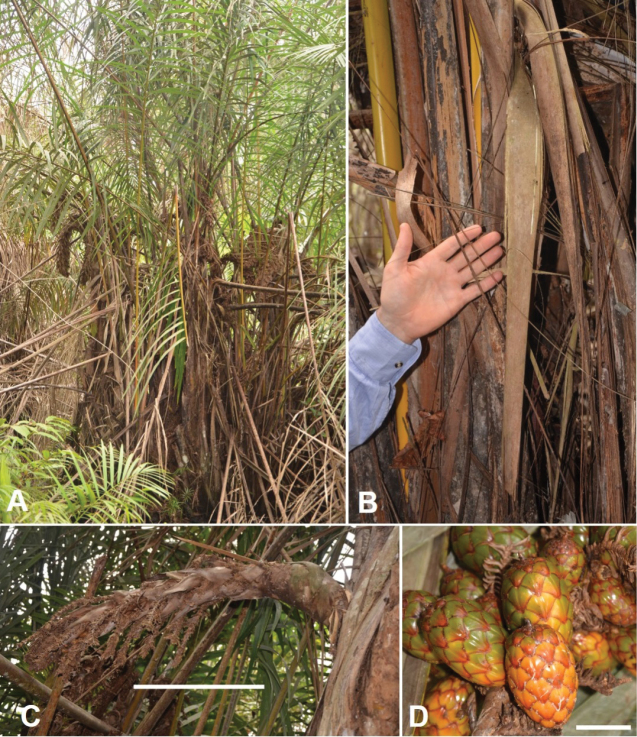
Morphology of *Raphia matombe*. **A**. Growth habit; **B**. Belt-like fibres downwards and the smallest fibres resulting from disintegration of the belt-like ones orientated in several directions, generally downwards; **C**. Young infructescence; **D**. Fruits. All from *Ndolo Ebika & Harris* 2739. Scale bars: 50 cm (**C**); 2.5 cm (**D**).

##### Ecology.

Swamps close to mangroves and edge of seasonally flooded watercourses in savannas. The species flowers in September and fruits in September and October.

##### Distribution.

Angola (including Cabinda), Democratic Republic of the Congo, and Republic of the Congo (Mbandu Luzolawo et al. 2020); in Republic of the Congo: Kouilou, Niari.

##### IUCN conservation assessment.

*Raphia matombe* was assessed by Cosiaux *et al*. (2018d) as Data Deficient (DD). The authors called for intensive surveys which will contribute to improving the species’ morphological description and better understanding of its distribution and threats.

##### Uses.

The sap is tapped and used as *Raphia* wine called Younga in Vili (Bas-Kouilou).

##### Vernacular names.

Mòkòngo in Vili and Loumbou (Bas-Kouilou, Kouilou); Konga in Vili (Ditadi, Niari).

##### Notes.

In the Republic of the Congo, this species seems to be restricted to an area within 100 km of the coast, but more fieldwork is required for a better understanding of the species’ distribution in the country. Stauffer et al. (2017) also suggested that it would be interesting to investigate in detail the growth form of this species as it was not known. The species is recognized by its petiole which is yellow and pale green when young and red when mature. This character also appears on the composite plate of the species in the Uíge Province in northern Angola as reported by Lautenschläger et al. (2019) on page 3365. In all our collections, *Raphia matombe* is the sole species recorded in swamps close to, but not in mangroves in the Republic of the Congo. Another species of *Raphia* growing in mangroves, *Raphia zamiana*, was described from Cameroon (Kamga et al. 2018). This species differs from *R. matombe* by its straight fibres covering the stipe and the lack of conspicuous and persistent bracts on the infructescence.

##### Specimen examined.

**Republic of the Congo**. • Kouilou. Bas-Kouilou, about 1 km SE of the bridge on the Kouilou River, 04°28.92'S, 011°43.40'E, 10 m, 27 Sep 2019, *Ndolo Ebika & Harris 2739* (HICPC, E); • Niari. Ditadi, 200 m NE of the road and 12 km SE of Dolisie, 04°16.24'S, 012°45.22'E, 400 m, 4 Oct 2019, *Ndolo Ebika & Harris 2761* (HICPC, E).

#### Raphia regalis

Taxon classificationPlantaeArecalesArecaceae

Becc. (Beccari 1910: 125)

668288FE-FD29-564B-8F57-993EB9D0BC95

Figs 15, 16

##### Description.

***Palms*** reaching 20 m high, solitary to gregarious. ***Stipe*** underground. ***Leaves*** arising directly from an underground stipe, 14.70–20.37 m long. Petiole 2.80–4.34 m long; DP30 between 6.3–9.4 cm. Rachis 11.90–16.32 m long, with two longitudinal rows of spines. Leaflets 135–175 × 5.8–8.2 cm, 2–8 in the R0–30 area of the rachis. ***Inflorescence*** 6 per individual, erect in early stages of development then hanging, brown then green, in the axil of a bract which has a long spine which is like an acumen. Partial inflorescence developing in zig-zag, not in a straight line. Secondary branches in the axil of small and persistent bracts, 4.5–5 × 0.5 cm. ***Flowers*** green in buds then dark red when open; Calyx with 3 lobes, acuminate. ***Fruits*** broadly ellipsoid, 6–7.2 × 2.8–3 cm, light brown when immature and dark brown when mature, with a 4 (–5) mm long beak.

**Figure 15.**
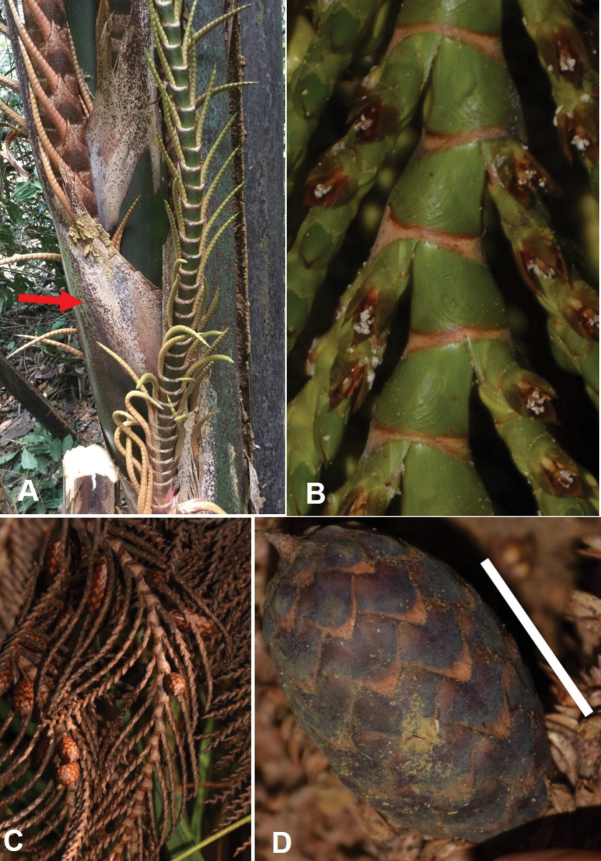
Morphology of *Raphia regalis*. **A**. Young inflorescences subtended by a bract (shown by the red arrow); **B**. Portion of an inflorescence with open female flowers; **C**. Young infructescence; **D**. Fruit. **A** from *Ndolo Ebika & Harris 2752***B** from *Ndolo Ebika & Harris 2753***C, D** from *Ndolo Ebika & Harris 2751*. Scale bar: 3 cm (**D**).

**Figure 16.**
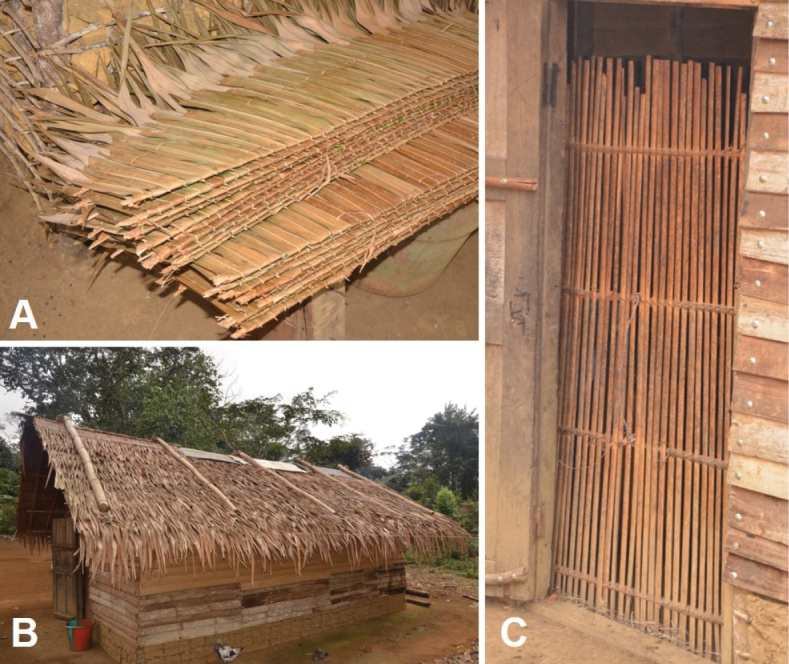
Use of *Raphia regalis*. **A**. Thatch roofing tiles; **B**. Roof covered with the thatch roofing tiles; **C**. Inner protecting door made from hard and smooth outer parts the petiole.

##### Ecology.

*Tierra firme*. The species flowers and fruits in October.

##### Distribution.

Angola (Cabinda), Cameroon, Gabon, Equatorial Guinea, Nigeria, Republic of the Congo, Democratic Republic of the Congo (Couvreur and Sunderland 2022; Mbandu Luzolawo et al. 2020; Mogue Kamga et al. 2019); in the Republic of the Congo: Lékoumou.

##### IUCN conservation assessment.

*Raphia regalis* was assessed by Cosiaux et al. (2018e) as Least Concern (LC).

##### Uses.

The petioles are used as laths to make the framework of huts, the skeleton of hut walls and also to make the two sticks that make up the skeleton which will be combined with the detached leaflets to form thatch roofing tiles.

##### Vernacular name.

Sougou in Téké (Lékoumou).

##### Notes.

The species is easily recognized by its leaves emerging straight from the ground. We observed around 15 individuals in an area of 400 m^2^ at Ingoumina.

##### Specimen examined.

**Republic of the Congo**. • Lékoumou. Ingoumina, 7 km from Zanaga on the road to Sibiti, 02°52.77'S, 013°52.39'E, 570 m, 1 Oct 2019, *Ndolo Ebika & Harris 2751* (HICPC, E); 7 km from Zanaga on the road to Sibiti, c. 3 km NE from Ingoumina village., 02°52.77'S, 013°52.39'E, 570 m, 1 Oct 2019, *Ndolo Ebika & Harris 2752* (HICPC, E); 7 km from Zanaga on the road to Sibiti, 02°52.83'S, 013°52.38'E, 570 m, 1 Oct 2019, *Ndolo Ebika & Harris 2753* (HICPC, E).

#### Raphia rostrata

Taxon classificationPlantaeArecalesArecaceae

Burret (Burret 1935: 307)

8B5C8E35-EE3D-57F0-9AD7-FECD8A6BBEE7

Fig. 17

##### Description.

***Palms*** 6–8 m high, gregarious. ***Stipes*** clustered, with 2 to 4 (–6) stipes joined at the base, covered with the leaf bases and fibres. Fibres at least 8 mm wide, curling, not forming springs around the stipe. ***Leaves*** 11.5–15.4 m long. Petiole 3.6–6.1 m long, reddish when very young, black with white dots to green when adult, channelled from the base to almost the middle then cylindrical or fully split, not spiny; DP30 comprised between 6.7–7.7 cm. Rachis 7.82–10.11 m long, not split or split in the first half then forming a single and prominent ridge. Leaflets 6–10 in the R0–30 zone, 159–186 × 4.8–6.6 cm. ***Infructescence*** 200 × 50 cm. ***Fruits*** oblong to cylindrical, rarely ellipsoid, 6–7.5 × 3–3.8 cm, reddish, with an 8–9 mm long beak.

**Figure 17.**
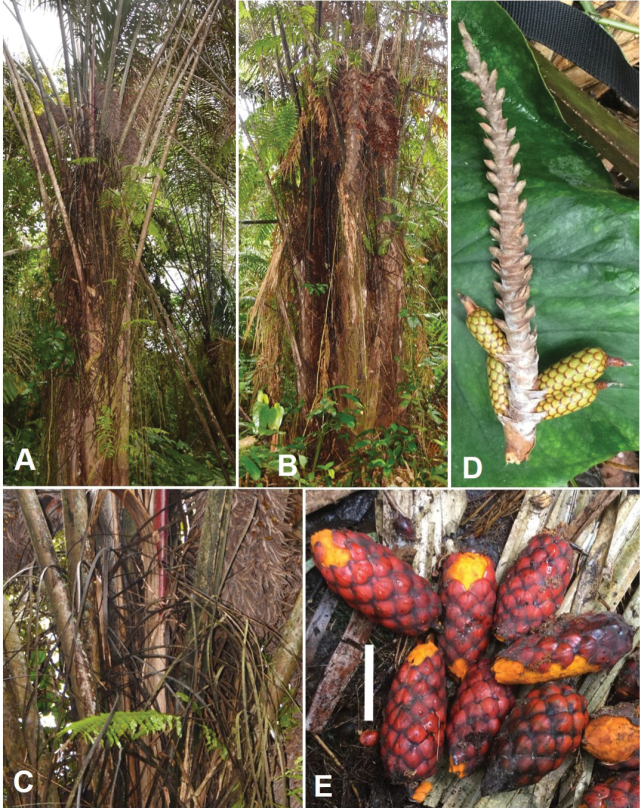
Morphology of *Raphia rostrata*. **A**. Solitary growth habit; **B**. Clustered habit; **C**. Upper part of the tree showing the stipe fibres; **D**. Partial infructescence with young fruits; **E**. Mature fruits. All from *Ndolo Ebika & Harris 2743*. Scale bar: 3.5 cm (**E**).

##### Ecology.

Swamp forest. The species fruits in September.

##### Distribution.

Angola (Cabinda), Cameroon, Gabon, Democratic Republic of the Congo (Couvreur and Sunderland 2022; Mbandu Luzolawo et al. 2020; Mogue Kamga et al. 2019), Republic of the Congo (this study); in Republic of the Congo: Lékoumou.

##### IUCN conservation assessment.

*Raphia rostrata* was assessed by Cosiaux et al. (2018f) as Data Deficient (DD). The authors requested more field surveys to better understand the taxonomic status, distribution, and threats of the species.

##### Use.

Production of *Raphia* wine called Touhomi in Téké (Ingoumina).

##### Vernacular name.

Touhomi in Téké (Lékoumou).

##### Notes.

*Raphia rostrata* resembles *Raphia hookeri* by the presence of curling stipe fibres. However, in *R. rostrata* the fibres are pendant and do not form tightly coiled springs whilst in *R. hookeri*, the fibres form tightly coiled springs which cover the stipe. In addition, the petiole is larger in *R. rostrata* with DP30 size of 6.7–7.7 cm. whereas the petiole of *R. hookeri* has a smaller diameter, with a DP30 size of 2.9–5.6 cm. Our single observation of *R. rostrata* was made in a swamp forest away from a stream whereas Couvreur and Sunderland (2022) reported from Cameroon that the species grows along fast flowing rivers.

##### Specimen examined.

**Republic of the Congo**. • Lékoumou. Ingoumina, 7 km from Zanaga on the road to Sibiti, c. 700 m W of the road, 02°53.23'S, 013°49.82'E, 540 m, 30 Sep 2019, *Ndolo Ebika & Harris 2743* (HICPC, E).

#### Raphia textilis

Taxon classificationPlantaeArecalesArecaceae

Welw. (Welwitsch 1859: 584)

AD2BD887-FC62-5074-A083-B6A8CBFB9050

Figs 18, 19

##### Description.

***Palms*** 6 to 10 m high, 16 cm in diameter, solitary. ***Stipes*** not clustered, with flattened, curling and hanging fibres, forming belt-like structures up to 4.5 cm wide and breaking down to form smooth fibres 5–6 mm wide. ***Leaves*** 3.78–12 m long. Petiole laterally fluted from base to rachis, not spiny, 1.55–4 m long; DP30 ranges between 5.0–5.1 cm. Rachis 2.23–8 m long, laterally fluted, with a row of spines on each side of the cleft. Base of rachis uneven, with an offset of 11 cm between the two groups of basal leaflets. Basal leaflets, on either side of the rachis cleft, grouped into a tuft of 4–8 leaflets, 86–96 × 3.4–5 cm, 32–33 in the R0-30 area of the rachis, with dorsal spines (on the main vein) 5 mm long and marginal spines 4 mm long. ***Inflorescences***: 3–4 per individual, hanging, about 2.5 m long. Partial inflorescence 23 × 13 cm. Secondary inflorescences 13–17 × 2 cm long. ***Male flowers***: corolla lobes 7–9 × 2 mm. ***Infructescence*** hanging, 3.80 m long. ***Fruits*** subglobose to obovoid, 5.3–6.5 × 4.8–5.2 cm, dark green when immature, becoming brownish and reddish when mature, truncated at the top with a 4 mm long beak.

**Figure 18.**
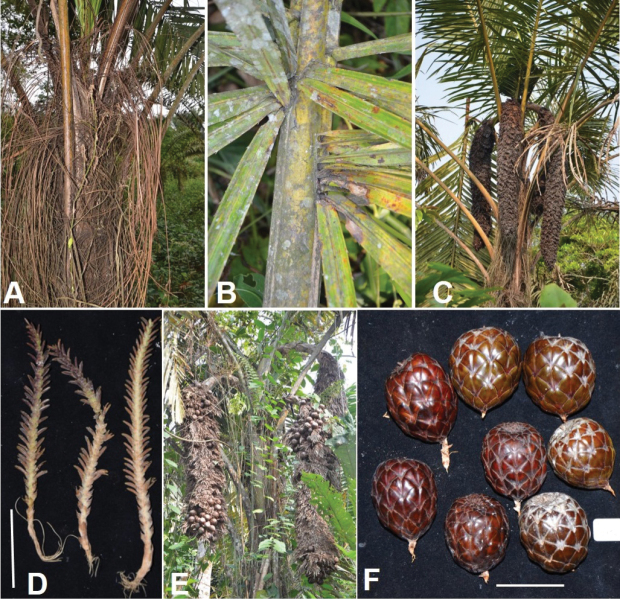
Morphology of *Raphia textilis*. **A**. Upper part of the stipe showing the fibres; **B**. Leaf portion showing an uneven base of rachis with an offset between the two groups of basal leaflets; **C**. Mature inflorescences, each about 2.5 m long; **D**. Secondary inflorescences; **E**. Infructescences; **F**. Fruits. All from *Ndolo Ebika 2702*. Scale bars: (**D, F**) 5 cm.

**Figure 19.**
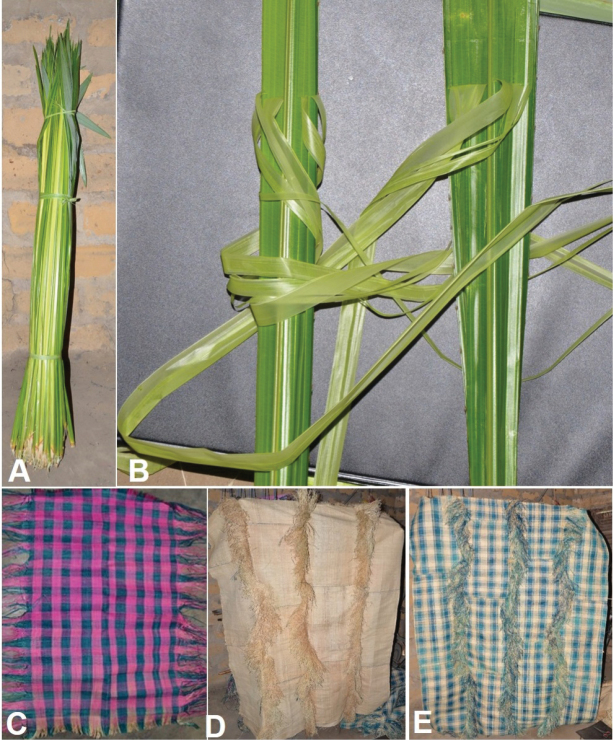
Use of *Raphia textilis*. **A**. Bunch of young leaflets, recently harvested; **B**. Epidermis removal from the young leaflets; **C**. Dyed, woven unit of Raphia cloth called “*Ketsoulou*” in Téké measuring 75 × 45 cm; **D**. Undyed composite piece of cloth made up from 13 of the *Ketsoulou* units shown in **C**; **E** dyed composite piece of cloth made up from 13 of the *Ketsoulou* units shown in **C**.

##### Ecology.

Growing on *tierra firme*. The species is cultivated in Lékana village (Plateaux) for commercial purposes. It flowers and fruits in August.

##### Distribution.

Angola (including Cabinda), Democratic Republic of the Congo, Gabon (Mogue Kamga et al. 2019; Tuley 1995), Republic of the Congo (this study); in the Republic of the Congo, the species occurs in Plateaux.

##### IUCN conservation assessment.

*Raphia textilis* has been assessed as Least Concern (LC) by Cosiaux et al. (2018g), who recommend further collecting efforts to better understand the area of occupancy of the species.

##### Uses.

The epidermis of the young leaflets of *Raphia textilis* is used to make raffia loincloths in Lékana (Plateaux).

##### Vernacular names.

Piki, plural Apiki, in Téké (Plateaux).

##### Specimens examined.

**Republic of the Congo**. • Plateaux. Lékana, 02°52.51'S, 013°50.06'E, 786 m, 13 Aug 2019, *Ndolo Ebika & Atikani 2702* (HICPC).

## Discussion

This study has increased our knowledge of the genus *Raphia* in the Republic of the Congo by bringing together new floristic and ecological data and analyses. This increase in knowledge is substantial, however, it is not yet complete, and a number of taxonomic and ecological questions still remain to be addressed.

Weconfirm the presence of seven species of *Raphia* in the Republic of the Congo. However, when we look at the taxonomic literature from neighboring countries (Gabon, Cameroon, and the Democratic Republic of the Congo) and from field and herbarium observations, we have some doubts about the application of names, which can only be resolved by a taxonomic revision. We present these doubts in the form of questions.

Question 1. What are the differences between *Raphia gentiliana* and *R. textilis*? *R. gentiliana* is not mentioned in “Flore du Gabon” (Mogue Kamga et al. 2019) and *R. textilis* is not mentioned in “Flore d’Afrique Centrale” (Mbandu Luzolawo et al. 2020), however we have recorded *R. gentiliana* and *R. textilis* from close to the border with Gabon in the west of Republic of the Congo and *R. gentiliana* across the country to the eastern border with the Democratic Republic of the Congo. Although Helmstetter et al. (2020) showed that the two species are different based on molecular evidence, we found it difficult to key out these taxa if the fruits are not available. In order to have enough morphological characters to facilitate the separation of these two species, it would be useful to make further observations on the two species from the Republic of the Congo and confirm whether there are gradients, or discontinuities in characters, and compare with both species from the surrounding countries.

Question 2. Are *R. hookeri* and *R. sese* sympatric or allopatric species? We have followed the concept of *R. hookeri* being a widespread and variable species across its distribution range from Sierra Leone to the Republic of the Congo. *R. hookeri* was treated in a similar way for Gabon (Mogue Kamga et al. 2019) and Cameroon (Couvreur and Sunderland (2022). We note however that *R. hookeri* is not recorded in “Flore d’Afrique Centrale” for the Democratic Republic of the Congo (Mbandu Luzolawo et al. 2020) and instead, a similar species, *R. sese*, is reported from the Democratic Republic of the Congo, with similar local names to the plant we are calling *R. hookeri* in the Republic of the Congo. Since our field observation on *R. hookeri* showed an overlap in the values of the length of the beak of the fruit compared to that of *R. sese* described in Mbandu Luzolawo et al. (2020) and Tuley (1995), we suggest that further investigations are required, making new observations and new collections to (a) improve the circumscription of these two species, which appear to display close genetic affinities (Helmstetter et al. 2020), (b) investigate the presence of *R. sese* in the Republic of the Congo, and (c) find if the two species share the same habitat or not.

Question 3. What is the distribution of *Raphia rostrata*? The type of this species is from Angola (Cabinda) and this palm is reported from the Democratic Republic of the Congo by Mbandu Luzolawo et al. (2020). From Cameroon and Gabon it is reported to be known only from a small number of collections and to grow along fast flowing rivers. This habitat differs from the one we observed in the Republic of the Congo. Field work needs to be conducted to collect material of this species to better understand its morphological variation, ecology, and distribution range.

Despite the efforts already made in documenting species, some complexes like *Raphia hookeri* and *Raphia laurentii* would benefit from some in-depth studies into species circumscription, distribution, ecology, and uses by people, especially in the Central African Republic, the Democratic Republic of the Congo, the Republic of the Congo, and Angola.

To encourage the engagement of non-palm specialists in *Raphia* research projects, we propose three innovations. Firstly, to provide simplified illustrations of the morphology of *Raphia*, to explain the morphology of the leaves, stipes, and especially the basic morphology of leaves, inflorescences, partial inflorescences, rachillae, bracts, and bracteoles. Even as taxonomists, we have struggled to match all of these terms consistently across descriptions and keys in the literature. Moreover, fragmentary herbarium specimens and lack of informative images continue hampering *Raphia* research and we believe that a simple illustrated glossary to the genus would help non-specialists to understand the different terms (Dransfield and Beentje 1996).

Our second proposal to improve the value of herbarium collections by people who are not *Raphia* specialists is to provide a checklist of observations that need to be recorded at the plant, which also acts as a list of photographs to be taken and a list of parts to be collected. This includes:

- Growth habit of the palm: whether it is solitary (with a single stipe); or clustered (with at least two stipes joined together at the ground level).
- Stipe: whether it is underground with all leaves emerging straight from the ground; or above ground.
- Stipe visibility: whether it is visible with leaves mostly located toward the top part of the palm; or invisible covered by the leaf sheaths or by old leaf sheaths, or by stipe fibres.
- Stipe fibre orientation and shape: whether they are straight and sharply pointed; or spring-like structures; or hanging and forming loose circles; or belt-like structures.
- Petiole measurements and shape: measurement of DP30 (diameter of the petiole at 30 cm below the first leaflet); length of petiole; and whether shape is circular or channelled in cross section.
- Rachis: how the leaflets are arranged at the base of the rachis (length of offset; or at the same level).
- Rachis shape: whether circular or channeled in cross section at the base.
- Rachis spines: whether present or not and position.
- Leaflets: number; length, and width.
- Inflorescence: orientation of the early-development stage (erect, suberect, or hanging); orientation at the mature stage; size and visibility of the bracts on the main axis.
- Fruits: size, shape, colour, and length of beak of immature and mature fruits.

Our third proposal is to create a list of vernacular standardized names for the Republic of the Congo in a similar way to pilot names used by the logging industry. Thus, instead of using *Entandrophragma cylindricum* (Sprague) Sprague and *Entandrophragma candolei* Harms, all actors, from administrator to chain saw operator, scientist and client buying timber, can all use the pilot names “sapelli” and “kosipo” with accuracy and precision. We suggest the following pilot names for *Raphia* species so far found in the Republic of the Congo based on the morphology, the local name, and the common use of the plant.

- «Raphia peke-molengue avec ressorts» *Raphia hookeri*.
- «Raphia à folioles décalées à la base et fruits longs » *Raphia gentiliana*.
- «Raphia tsam à longues fibres pointues » *Raphia laurentii*.
- «Raphia à pétiole tricolore » *Raphia matombe*.
- «Raphia à gros pétiole tacheté de blanc » *Raphia rostrata*.
- «Raphia des pagnes avec des fruits ronds» *Raphia textilis*.
- «Raphia géant sans tronc » *Raphia regalis*.

The ecological importance of *Raphia* was noted but not studied during this research. We deemed it necessary to first correctly identify the species before studying their ecology. Each species we found appeared to thrive in a particular habitat different from that of other *Raphia* species in what appeared to be naturally occurring populations.

Field observations we made across the country allowed us to record some threats to the habitats of *Raphia* species. We saw evidence of large-scale dieback of *R. hookeri* in swamps close to roads which had recently been upgraded. This can be due to the fact the species is mainly growing beside fast flowing rivers. By modifying the current of the river, this leads to a change of the water level in the adjacent swamps, eventually leading to the death of the palms. We think that a study on the effect of road construction or improvement on *Raphia* dominated swamps should be carried out urgently to understand the impact of such activities and what mitigation actions might be required. In addition, the cutting down of palms for the harvesting of larvae of *Rhynchophorus cf. phoenicis* represents a threat to palm species growing in low density in specific areas.

## Conclusion

The study conducted on the genus *Raphia* in the Republic of the Congo allowed us to document the occurrence of seven species: *R. gentiliana* De Wild., *R. hookeri* G.Mann & H.Wendl., *R. laurentii* De Wild., *R. matombe* De Wild., *R. regalis* Becc., *R. rostrata* Burret and *R. textilis* Welw. in the country. *Raphia rostrata* is reported here for the first time. Of these species, *R. regalis* is distinguished from the other species by having an underground stipe. *R. laurentii* was the only species recorded with straight and upwards-orientated stipe fibres in addition to being a clustered palm like *R. matombe* and, in some extent, *R. rostrata*. *Raphia hookeri* was characterized by a stem covered with fibres of at least 8 mm width which form dense springs. Two species, *R. rostrata* and *R. matombe*, showed variation of the petiole’s colour between the young and mature stage. The petiole is reddish when very young then black with white dots to green when adult in *R. rostrata*; yellow to pale green when young and red when mature in *R. matombe*. To standardize the measurements of the petiole’s diameter across the *Raphia* species, we proposed taking the diameter of the petiole at 30 cm below the first leaflet, which was referred to as DP30, as this area of the petiole close to the leaflets is easily accessible on the leaf regardless of the length at which the leaf was cut off the from the stipe.

The informal interviews combined with direct observations allowed us to record the local names of each *Raphia* species and the uses people make of parts of the *Raphia* plant. Two species, *R. gentiliana* and *R. textilis*, are used for clothing. The epidermis of young leaflets of *R. gentiliana* is used to make traditional dance skirts whereas in *R. textilis* it is used to make pieces of cloth. The sap is tapped and used to make *Raphia* wine from four species: *R. gentiliana*, *R. hookeri*, *R. laurentii*, and *R. matombe*. Species such as *R. laurentii*, *R. regalis* and to some extent, *R. hookeri*, provide materials for building huts.

This study provides a baseline for *Raphia* species in the Republic of the Congo. We suggest that by carrying out botanical inventories and ethnobotanical surveys in areas not covered by the current work, it will be possible to find more species and other material uses of *Raphia* spp.

## Supplementary Material

XML Treatment for Raphia gentiliana

XML Treatment for Raphia hookeri

XML Treatment for Raphia laurentii

XML Treatment for Raphia matombe

XML Treatment for Raphia regalis

XML Treatment for Raphia rostrata

XML Treatment for Raphia textilis

## Additional information

### Use of AI

No use of AI was reported.

### Author ORCIDs

Sydney T. Ndolo Ebika https://orcid.org/0000-0002-0933-6948

Gaël U. D. Bouka https://orcid.org/0000-0001-8850-522X

David J. Harris https://orcid.org/0000-0002-6801-2484

Raymond G. Elenga https://orcid.org/0000-0002-8115-396X

### Data availability

All of the data that support the findings of this study are available in the main text or Supplementary Information.

## Supplementary materials

Supplementary material 1 Supplementary information Data type csv Explanation note A file containing specimens and measurements used in morphometric data matrix construction and analyses. https://binary.pensoft.net/file/1536724This dataset is made available under the Open Database License (http://opendatacommons.org/licenses/odbl/1.0/). The Open Database License (ODbL) is a license agreement intended to allow users to freely share, modify, and use this Dataset while maintaining this same freedom for others, provided that the original source and author(s) are credited. Sydney T. Ndolo Ebika, Gaël U. D. Bouka, David J. Harris, Raymond G. Elenga
